# Alterations of the Gut Microbiota in Multiple System Atrophy Patients

**DOI:** 10.3389/fnins.2019.01102

**Published:** 2019-10-18

**Authors:** Linlin Wan, Xin Zhou, Chunrong Wang, Zhao Chen, Huirong Peng, Xuan Hou, Yun Peng, Puzhi Wang, Tianjiao Li, Hongyu Yuan, Yuting Shi, Xiaocan Hou, Keqin Xu, Yue Xie, Lang He, Kun Xia, Beisha Tang, Hong Jiang

**Affiliations:** ^1^Department of Neurology, Xiangya Hospital, Central South University, Changsha, China; ^2^National Clinical Research Center for Geriatric Disorders, Central South University, Changsha, China; ^3^Key Laboratory of Hunan Province in Neurodegenerative Disorders, Central South University, Changsha, China; ^4^Laboratory of Medical Genetics, Central South University, Changsha, China; ^5^Department of Neurology, Xinjiang Medical University, Urumchi, China

**Keywords:** multiple system atrophy, microbiota, metagenomics, functional pathways, inflammation

## Abstract

Multiple system atrophy (MSA) is a fatal neurodegenerative disease, and the pathogenesis is still quite challenging. Emerging evidence has shown that the brain–gut–microbiota axis served a pivotal role in neurological diseases; however, researches utilizing metagenomic sequencing to analyze the alteration in gut microbiota of MSA patients were quite rare. Here, we carried out metagenomic sequencing in feces of 15 MSA patients and 15 healthy controls, to characterize the alterations in gut microbial composition and function of MSA patients in mainland China. The results showed that gut microbial community of MSA patients was significantly different from healthy controls, characterized by increased genus *Akkermansia* and species *Roseburia hominis*, *Akkermansia muciniphila*, *Alistipes onderdonkii*, *Streptococcus parasanguinis*, and *Staphylococcus xylosus*, while decreased genera *Megamonas*, *Bifidobacterium*, *Blautia*, and *Aggregatibacter* and species *Bacteroides coprocola*, *Megamonas funiformis*, *Bifidobacterium pseudocatenulatum*, *Clostridium nexile*, *Bacteroides plebeius*, and *Granulicatella adiacens*. Further, functional analysis based on the KEGG database revealed aberrant functional pathways in fecal microbiome of MSA patients. In conclusion, our findings provided evidence for dysbiosis in gut microbiota of Chinese MSA cohorts and helped develop new testable hypotheses on pathophysiology of MSA.

## Introduction

Multiple system atrophy (MSA) is a sporadic, adult-onset, progressive neurodegenerative disease, characterized by diverse combinations of parkinsonian features, cerebellar ataxia, autonomic failure, and pyramidal features. Clinically, MSA is divided into two subtypes: MSA with predominant parkinsonism (MSA-P) and MSA with predominant cerebellar ataxia (MSA-C) (Fanciulli and Wenning, 2015). The typical neuropathology of MSA is oligodendroglial cytoplasmic inclusions (GCIs), mainly composed of α-synuclein (Papp et al., 1989; Gai et al., 1998). Till now, environmental factors (such as nicotine use and alcohol consumption) and genetic factors (such as mutations in COQ2, SHC2, and SNCA) are thought to contribute to the risk of MSA together. However, the explicit etiology and pathogenic mechanisms remain unclear (Fanciulli and Wenning, 2015). Due to the variable manifestations, the diagnostic accuracy of MSA remains a challenge. The definite diagnosis of MSA requires the neuropathologic evidence of widespread α-synuclein-positive glial cytoplasmic inclusions along with olivopontocerebellar atrophy or striatonigral degeneration in postmortem examination (Trojanowski and Revesz, 2007). MSA is a rare severe neurodegenerative disease with 6- to 10-year mean survival time from symptom onset; however, there is no cured therapy yet and only symptomatic treatment is available, which is also quite disappointing (Flabeau et al., 2010; Fanciulli and Wenning, 2015).

Microbiota, defined as the entirety of microorganisms in a specific habitat, is gaining increasing importance (Whiteside et al., 2015). It has been shown to serve pivotal roles in numerous diseases, such as obesity, cardiovascular diseases, dermatosis, hepatic diseases, and osteoarticular diseases (Collins et al., 2015; Li et al., 2016, 2017; Liu et al., 2017; Tan L. et al., 2018; Zhou et al., 2019). The brain–gut–microbiota axis has put forward a new promising direction for neuroscience. The gut microbiota could interact with brain through immune, neural, and neuroendocrine pathways to regulate brain development, function, and behavior (Dinan and Cryan, 2017). Therefore, microbiota may serve pivotal roles in pathogenesis of neurological diseases, which has been proven in Parkinson’s disease, myasthenia gravis, multiple sclerosis, etc. (Keshavarzian et al., 2015; Jangi et al., 2016; Qiu et al., 2018). As for MSA, intestinal inflammation has been suggested to be related with MSA pathogenesis (Cao et al., 2018); thus, the gut microbiota may also participate in pathogenesis of MSA. Engen et al. (2017) discovered the alteration of gut microbiota in American MSA patients through 16SrDNA sequencing in feces and sigmoid mucosa. Tan et al. used 16SrDNA sequencing, revealing that the fecal microbiota was different between MSA patients and healthy persons in ethnic Chinese of Malaysia (Tan A.H. et al., 2018). The studies focusing on the change in gut microbiota of MSA patients were quite rare.

Traditionally, 16SrDNA sequencing is the main approach for investigating the microbiota based on amplifying and sequencing the hypervariable loci in 16SrDNA gene of bacteria (18SrDNA for fungi). This method requires low cost and input template DNA concentrations; however, it cannot characterize bacteria to their species/strain levels and is limited when used in analyzing the functions of the microbiota. Metagenomics is defined as sequencing the entire DNA (or RNA) content of microbiota in a sample through high-throughput techniques. Metagenomics, besides identifying microbial taxa to their species/strain levels, possesses more superiority in analyzing the function of genes, the structure and organization of genomes, community structure and evolutionary relationships within the sample, and identification of new genes and biocatalysts (Cao et al., 2017; Martinez et al., 2017; Roumpeka et al., 2017). However, there were no researches utilizing metagenomic sequencing to analyze the relationships between gut microbiota and MSA pathogenesis yet. Here, we carried out metagenomic sequencing to compare the microbiota community in feces of MSA patients to healthy controls of mainland China and further explore the key alterations of functional pathways in MSA patients’ gut microbiota.

## Materials and Methods

### Subjects

Multiple system atrophy patients (*n* = 15; 8 males, 7 females; mean age 56.7 ± 8.4 years) were recruited from the Department of Neurology of Xiangya Hospital; meanwhile, healthy control volunteers (*n* = 15; 10 males, 5 females; mean age 53.8 ± 7.5 years) were recruited from the Physical Examination Center of Xiangya Hospital. The study was approved by the Ethics Committee of Xiangya Hospital of Central South University in China. All subjects gave written informed consent in accordance with the Declaration of Helsinki.

Multiple system atrophy patients were diagnosed by at least two neurologists according to the second consensus statement of diagnostic criteria for MSA (Gilman et al., 2008). SCA (spinocerebellar ataxia) 1, 2, 3, 6, 7, and 17 and DRPLA (dentatorubral-pallidoluysian atrophy) were genetically excluded. The inclusion criteria of healthy control group subjects were as follows: (1) age, sex, and BMI were matched to MSA groups; (2) no constipation and diarrhea; and (3) no neurological diseases. Exclusion criteria for both group subjects were as follows: (1) the use of antibiotics, glucocorticoids, probiotics, and immunosuppressants within the 2 months before the sample collection; (2) hypertension, diabetes, obesity, and metabolic syndrome; and (3) gastrointestinal diseases and autoimmune diseases. Besides, both group subjects were from the Han nationality in central south area of China.

Clinical information of all subjects was collected through face-to-face interviews between subjects and neurological specialists. All subjects finished a food frequency questionnaire (FFQ) through recalling the dietary frequency in the period of 1 year before the interview. Besides, we especially recorded the intake of the fish oil in the dietary supplement, since the unsaturated fatty acid could affect the gut microbiota composition (Zmora et al., 2019). In addition, we calculated the dietary diversity score (DDS) of each subject based on the FFQ results. According to the Chinese Dietary Guidelines, all food items were categorized into nine groups: grains, vegetables, fruit, meat, fish, eggs, beans, dairy, and oil (Zhang et al., 2017). If the participant ate any food from these nine categories at least once a week, one point was given in that category. Otherwise, the score was zero. Consuming different food in the same category would not count repeatedly. The DDS was equal to the sum points of the nine categories. The symptoms of MSA subjects were assessed through the Unified Multiple System Atrophy Rating Scale (UMSARS) and the cognitive status was evaluated by the Mini Mental State Examination (MMSE). The constipation of all subjects was assessed by the Wexner constipation score.

### Sample Collection and DNA Extraction

Each MSA patient and healthy subject provided a fresh stool sample in the morning using a fecal collection container, which was immediately transported on ice to the laboratory and preserved at −80°C. QIAamp Fast DNA Stool Mini Kit (Qiagen, Germany) was used to extract DNA from fecal samples. The DNA integrity and concentration were tested by 1% agarose gel electrophoresis (AGE).

### Metagenomic Sequencing

#### DNA Library Construction and Sequencing

The metagenomic libraries were constructed by using KAPA Hyper Prep Kit (Kapa Biosystems, United States) according to manufacturer’s instructions, with an average of 350 bp insert size. The quality was assessed on a Qubit@ 2.0 Fluorometer (Thermo Scientific, United States) and the Agilent Bioanalyzer 2100 system (Agilent, United States). Sequencing was performed on an Illumina HiSeq2500 platform.

#### Bioinformatics and Statistical Analysis

Reads that mapped to human genome (hg19) were removed from raw reads and then low-quality reads plus reads with adaptors were filtrated through Trimmomatic software (version 0.38). The quality of Illumina raw reads and clean reads was analyzed by using FastQC software (version 0.11.8). Species taxonomic assignment was achieved through aligning clean reads to clade-specific marker genes available on NCBI using MetaPhlAn2 software (version 2.0). Partial least square-discriminant analysis (PLS-DA) was performed through R software (version 3.4.1). LDA effect size (LEfSe) analysis was utilized to identify bacterial taxa with significantly different abundance between MSA and healthy groups based on LEfSe software (version 1.0). The alpha cutoff was set to 0.05 and effect size cutoff was set to 2.0. Because LEfSe required all the pairwise comparison to reject the null hypothesis to detect a biomarker, no multiple testing corrections were needed (Segata et al., 2011). Differential abundance analysis between MSA-C and MSA-P subtypes was performed through the Welch’s *t*-test and *p-*values were corrected through Storey’s false discovery rate (FDR) control. An FDR *q* value < 0.05 was considered statistically significant.

*De novo* assembly was carried out through Megahit software (version 0.3.0) to obtain contigs (>500 bp) with five k-mer parameters 21, 41, 61, 81, and 99. We use Prodigal software (version 2.6.3) to perform gene (open reading frame, ORF) prediction on contigs from each sample (genes shorter than 100 bp were removed) and translation to protein sequences. Then CD-HIT software (version 4.6.8) was utilized to cluster predicted genes and redundant sequences were removed based on 95% identity and 90% coverage. The longest sequence of each cluster was selected to construct non-redundant gene catalog and the final non-redundant gene catalog contains 1,925,921 microbial genes. Next, we utilized Kallisto software (version 0.44.0) to calculate gene reads counts and perform normalization on reads counts to obtain relative gene abundance through the TMM method implemented in edgeR software (version 3.14.0). Functional annotations were carried out through DIAMOND software (version 0.8.22) against the KEGG database (Kanehisa et al., 2004) and dbCAN database (Yin et al., 2012) (*e* value≤1e−5). Then, we calculated the abundance of each functional feature by summing the abundance of genes annotated to this feature. For functional features with an abundance median≥1e−8 in both groups, differential abundance analysis was performed using the Welch’s *t*-test in STAMP software (version 2.1.3). *p-*values were corrected through Storey’s FDR method and we applied a cutoff of FDR *q* value < 0.1 to indicate results remaining noteworthy. Demographic and clinical characteristics were compared with Student’s *t*-test, Fisher exact test, and Mann–Whitney *U* test for quantitative and categorical variables, based on the SPSS software (version 19.0).

## Results

### The Basic Characteristics of Subjects

The demographic and clinical characteristics of subjects are summarized in Table 1. In total, 15 MSA patients and 15 healthy controls were recruited in bioinformatic analysis. There was no difference observed in age, gender, and BMI between two groups. As for the diet pattern, no significant difference was detected in all food category consumption and dietary diversity scores. No subject consumed the fish oil as the dietary supplement (Supplementary Table S1). MSA patients including 11 MSA-C subtypes and 4 MSA-P subtypes had an average disease duration of 2.3 ± 0.8 years, mean UMSARS I scores of 19.7 ± 5.2, mean UMSARS II scores of 21.2 ± 6.9, mean UMSARS IV scores of 3.6 ± 0.7, and average MMSE scores of 26.3 ± 2.4. Besides, MSA patients had higher Wexner constipation scores than healthy controls (7.3 vs. 1.2, *p*-value < 0.001).

**TABLE 1:** Baseline demographic and clinical characteristics of subjects.

**Measure**	**MSA group**	**Healthy group**	***p-*value**
*n*	15	15	–
Age (years)^∗^	56.7 (8.4)	53.8 (7.5)	0.332
Disease duration (years)^∗^	2.3 (0.8)	–	–
Female/male	7/8	5/10	0.710
BMI (kg/m^2^)^∗^	22.4 (2.4)	22.8 (1.5)	0.533
Subtype (C/P)	11/4	–	–
Wexner constipation scores^∗^	7.3 (5.2)	1.2 (0.9)	<0.001
UMSARS I scores^∗^	19.7 (5.2)	–	–
UMSARS II scores^∗^	21.2 (6.9)	–	–
UMSARS IV scores^∗^	3.6 (0.7)	–	–
MMSE scores^∗^	26.3 (2.4)	–	–

*^∗^Presented as mean (SD); “–”, not applicable or not available; MSA, multiple system atrophy; BMI, body mass index; C, MSA-C subtype; P, MSA-P subtype; UMSARS, unified multiple system atrophy rating scale; MMSE, mini mental state examination.*

### Taxonomy Annotation and Microbiota Composition

**FIGURE 1:**
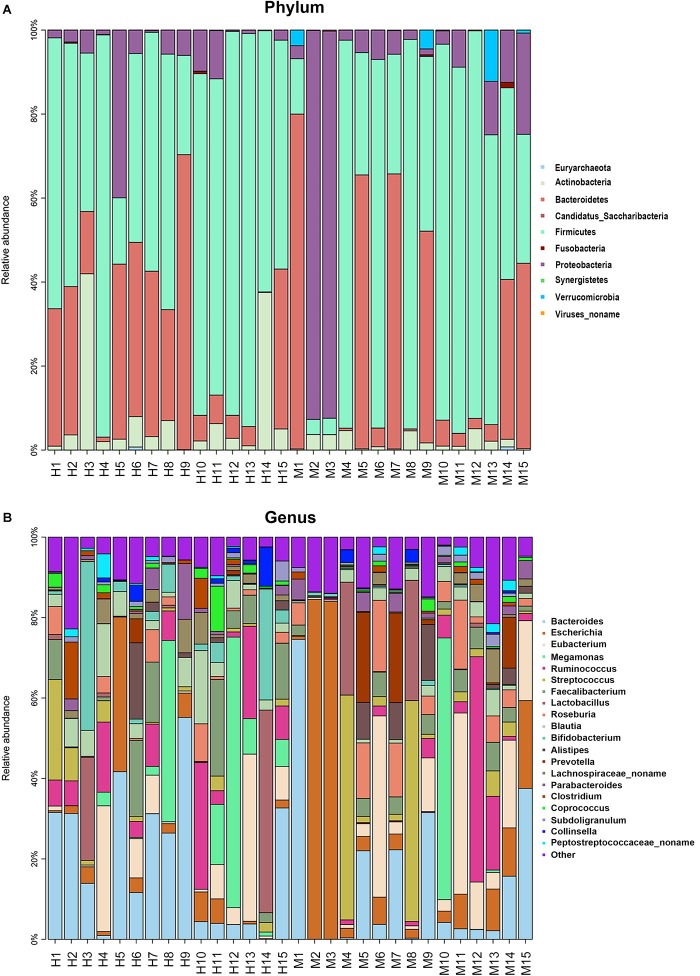
The microbiota composition of each sample. Each bar represented different bacterial taxon and the length represented the relative abundance. **(A)** The relative abundance of top 10 taxa on phylum level. **(B)** The relative abundance of top 20 taxa on genus level. H, healthy group; M, MSA group.

**FIGURE 2:**
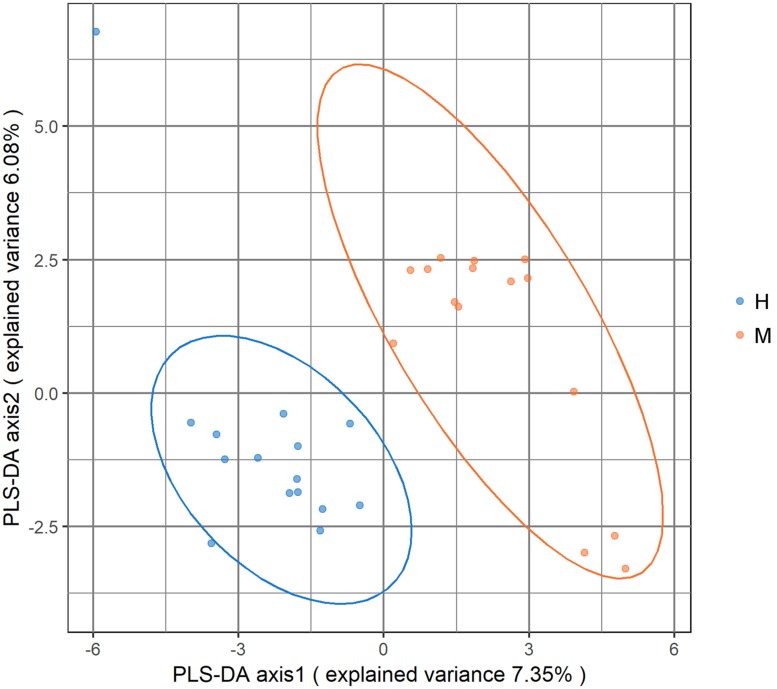
Score plot of partial least square-discriminant analysis (PLS-DA) showed that the MSA and healthy groups were separated into two clusters. Each dot represented one sample.

Metagenomic sequencing resulted in an average of 37.28 ± 3.56 million clean reads each sample. The specific quality of clean reads in each sample was shown in Supplementary Table S2. After taxonomy annotation based on NCBI, we summarized the microbiota composition of each sample on phylum (top 10) and genus (top 20) levels in Figure 1. In the stacked bar chart, each bar represented different bacterial taxon and the length represented the relative abundance. The results showed that Firmicutes, Bacteroidetes, and Proteobacteria were dominant phyla in both MSA and healthy groups. The samples were then analyzed with PLS-DA on the genus level. The score plots revealed a clear discrimination of microbial profile between MSA and healthy control groups (Figure 2), which reflected the alterations in MSA patients’ gut microbial community.

### Differential Microbial Taxa Analysis

**FIGURE 3:**
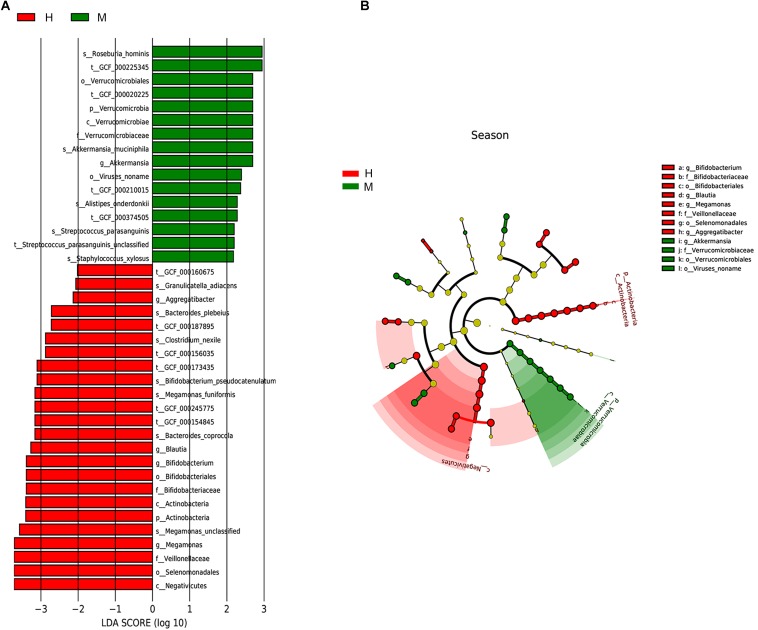
Taxonomic differences of gut microbiota in MSA and healthy groups predicted on different levels. **(A)** Histogram of the LDA scores computed for taxa differentially abundant between groups. **(B)** Cladogram indicated the phylogenetic distribution of fecal microbiota associated with MSA and healthy subjects. Each dot represented a taxonomic entity and the taxonomic levels ranged from phylum to species from the inner to outer circles.

We utilized the LEfSe method to elucidate which taxa were driving divergence between MSA and healthy groups. We set the LDA score cutoff as 2.0 to distinguish the significant bacterial difference on different taxonomic levels. The results are illustrated in Figure 3 and showed a remarkable difference in gut microbiota between groups (Figures 3A,B). The phylum *Verrucomicrobia* was increased whereas phylum Actinobacteria was decreased in MSA patients. At the genus level, MSA group possessed more abundant *Akkermansia*, while *Megamonas*, *Bifidobacterium*, *Blautia*, and *Aggregatibacter* were more abundant in the healthy group. Further, to analyze at the species level, the relative abundance of *Roseburia hominis*, *Akkermansia muciniphila*, *Alistipes onderdonkii*, *Streptococcus parasanguinis*, and *Staphylococcus xylosus* in the MSA group was higher than those in the healthy group, while the healthy group had more abundant *Bacteroides coprocola*, *Megamonas funiformis*, *Bifidobacterium pseudocatenulatum*, *Clostridium nexile*, *Bacteroides plebeius*, and *Granulicatella adiacens*. In addition, we did not detect the differential taxa at an FDR *q* value cutoff of 0.05 between MSA-C and MSA-P subtypes. Since only four MSA-P patients were included in our study, differential microbiome analysis between MSA subtypes needs further verification in larger samples.

### Gene Prediction and Non-redundant Gene Catalog Construction

After *de novo* assembly, we obtained contigs with a mean number of 105.97 ± 47.78 thousand in each sample and the assembly result of each sample was illustrated in Supplementary Table S3. Then, we performed prediction on contigs to gain gene and protein sequences. The distribution of gene length in each sample is shown in Supplementary Figure S1 and Supplementary Table S4. After clustering on predicted genes, we selected the longest sequence of each cluster to construct a non-redundant catalog, and the final non-redundant gene catalog contained 1,925,921 microbial genes.

### Differential Functional Pathways Analysis

**FIGURE 4:**
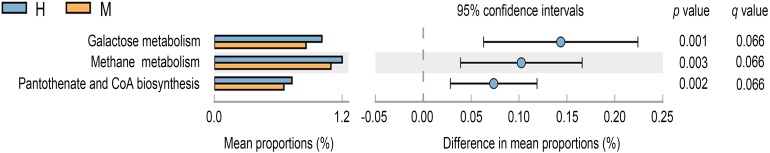
Differential KEGG pathways at level 3 for the fecal microbiome of MSA and healthy groups. Mean proportions are shown in stacks for MSA (orange) and healthy (blue) groups. Difference in mean proportions = mean proportions in healthy group minus mean proportions in MSA groups. *q* values were calculated through Storey’s FDR method.

To further investigate significantly altered functions between MSA patients and healthy controls’ gut microbiota, we annotated the gene catalog against the KEGG database and dbCAN database. Based on the KEGG database, the gene counts under each functional pathway were illustrated in Supplementary Figure S2 and the metabolism function catalog was with the most genes distributed. As illustrated in Figure 4 (*p*-value < 0.05, FDR *q* value < 0.1), the microbial gene functions including galactose metabolism (*p*-value = 0.001, FDR *q* value = 0.066), methane metabolism (*p*-value = 0.003, FDR *q* value = 0.066), and pantothenate and CoA biosynthesis (*p*-value = 0.002, FDR *q* value = 0.066) were much higher in fecal microbiome of the healthy group. In addition, a vital role of the human intestinal microbiota in hosts is to metabolize the dietary glycans and the carbohydrates of the host mucus (Koropatkin et al., 2012). Carbohydrate-active enzymes (CAZymes) encoded by the gut microbiota are responsible for synthesis, degradation, and modification of carbohydrates (Yin et al., 2012). The distribution of gene counts annotated under different CAZyme terms is shown in Supplementary Figure S3. However, there were no significantly differential CAZyme terms detected at an FDR cutoff of 0.1 between two groups.

## Discussion

Our study showed that MSA patients had a distinction in taxa composition and function of gut microbiota compared to healthy controls based on metagenomic sequencing of fecal samples. Although fecal microbiota may not reflect gut bacterial communities exactly, metagenomic sequencing based on stool could still be informative. It contained bacteria from mucosal desquamation and was the easiest sampling technique up to the standard of related investigations (Durban et al., 2011; Vazquez-Castellanos et al., 2015). Our study revealed that MSA patients presented a distinctive microbiota composition compared to healthy controls, characterized by high abundant genus *Akkermansia* and species *R. hominis*, *A. muciniphila*, *A. onderdonkii*, *S. parasanguinis*, and *S. xylosus*, while low abundant genera *Megamonas*, *Bifidobacterium*, *Blautia*, and *Aggregatibacter* and species *B. coprocola*, *M. funiformis*, *B. pseudocatenulatum*, *C. nexile*, *B. plebeius*, and *G. adiacens*. Functional analysis based on the KEGG database showed that pathways involving galactose metabolism, methane metabolism, and pantothenate and CoA biosynthesis were less abundant in fecal microbiome of MSA patients. In dbCAN database, we did not detect the significantly differential CAZyme terms between two groups.

Multiple system atrophy is a fatal neurodegenerative disease as well as synucleinopathy, with challenges in pathogenesis explication, diagnosis, and therapy. Recently, increasing evidence has shown that alteration of the gut microbiota could influence neural development, cognition, and behavior via the bidirectional interaction with the brain–gut–microbiota axis (Rogers et al., 2016). In another synucleinopathy, Parkinson’s disease, 16SrDNA sequencing of sigmoid mucosal and fecal samples revealed that *Ralstonia*, *Akkermansia*, *Oscillospira*, and *Bacteroides* were more abundant while *Blautia*, *Coprococcus*, *Roseburia*, and *Faecalibacterium* were less enriched in Parkinson’s disease patients (Keshavarzian et al., 2015). Besides, colonization with microbiota from Parkinson’s disease patients was found to exacerbate physical impairments in germ-free transgenic Parkinson’s disease mice (Sampson et al., 2016). As for MSA, Engen et al. found that at the family level, the abundance of *Clostridiaceae* and *Rikenellaceae* were higher while *Lachnospiraceae* (genera *Ruminococcus*, *Roseburia*, and *Coprococcus*) and *Ruminococcaceae* (genus *Faecalibacterium*) were lower in American MSA patients’ fecal sample (Engen et al., 2017). Tan et al. found that in ethnic Chinese of Malaysia, MSA patients had more abundant *Bacteroides* and less abundant *Paraprevotella* in fecal microbiota at the genus level (Tan A.H. et al., 2018). Both two studies were based on 16SrDNA sequencing.

Among different bacterial taxa based on our study, genus *Akkermansia* was also found to be increased in gut microbiota of Parkinson’s disease patients (Keshavarzian et al., 2015; Petrov et al., 2017). *Akkermansia* could produce short-chain fatty acids (SCFAs). SCFAs had several beneficial effects on host: maintaining epithelial barrier function, diminishing oxidative DNA damage, regulating cytokine production, anti-inflammatory effects, and stimulating immune function (Maslowski and Mackay, 2011). However, *Akkermansia* was reported to have proinflammatory properties, upregulating genes involved in antigen presentation pathway, B and T cell receptor signaling, IL-4 signaling, and complement and coagulation cascades (Derrien et al., 2011). These proinflammatory features may be due to its ability to disturb host mucus homeostasis, resulting in the breakdown of the gut barrier (Ganesh et al., 2013). Inflammation served a critical part in MSA pathogenesis (Fanciulli and Wenning, 2015; Zhou et al., 2018) and gut inflammation was shown to increase the risk of MSA. Besides, some gut-inflammation risk genes could also increase the risk of MSA, such as LRRK2 and NOD2; therefore, putative proinflammatory bacterial genera may participate in stimulating MSA process (Franke et al., 2010; Heckman et al., 2014; Engen et al., 2017; Cao et al., 2018; Villumsen et al., 2019). As for reduced bacteria, *Blautia* was a butyrate-producing genus (Keshavarzian et al., 2015) and butyrate was the most pronounced SCFA, which had anti-inflammation properties (Maslowski and Mackay, 2011; Pirozzi et al., 2018). Genus *Bifidobacterium* was also an inflammation-suppressing bacteria, and bioactive factors from *Bifidobacterium* were shown to improve epithelial cell barrier resistance and, thus, attenuated inflammation (Ewaschuk et al., 2008), which hinted the suppressive influence of Bifidobacterium for MSA. However, the detailed roles of these different bacterial taxa in pathogenesis of MSA still need further exploration. The bacterial alteration in MSA patients of mainland China identified from our study was different from that in American and ethnic Chinese of Malaysia (Engen et al., 2017; Tan A.H. et al., 2018). Numerous factors may be responsible for the discrepancy. First, there is the inherent discrepancy between 16SrRNA and metagenomic sequencing, which was reported previously in other studies (Shah et al., 2011; Poretsky et al., 2014; Hong et al., 2016). Then, bias due to the small sample size could not be excluded.

Our functional analysis revealed the aberrant functional pathways of MSA patients’ gut microbiota based on the KEGG database, which were all related with metabolism. Galactose metabolism was attached to the carbohydrate metabolism, and methane metabolism was related to the energy metabolism. Moreover, pantothenate and CoA biosynthesis belonged to the metabolism of cofactors and vitamins. The results revealed the aberrant metabolism in MSA patients’ gut microbiota. The host–microbe metabolic axis is a multi-directional interactive chemical highway between specific host and microbe functional pathways. The gut microbiota could produce a large array of metabolites that are necessary for host health, such as bile acids, choline, SCFAs, aromatic amino acids, etc. Microbe-produced metabolites could affect host metabolic phenotype, energy homeostasis, immune, inflammation, etc., and hence regulate disease process (Nicholson et al., 2012). Energy failure and inflammation are significant contributors to MSA pathogenesis (Jellinger, 2014). For more details about the functional alteration in gut microbiota of MSA patients and its roles in the MSA process, multi-omics combination including metabonomics, transcriptomics, proteomics, etc., was required. In addition, a function experiment based on the germ-free mice was also necessary.

There were still big challenges in MSA pathogenesis interpretation, precise diagnosis, and efficient treatment. Going into the roles of gut microbiota in MSA may shed light on its pathogenesis. Besides, the bacterial taxa with differential abundance between MSA patients and healthy persons might be utilized as novel biomarkers, contributing to the clinical diagnosis. In addition, rebalancing the gut microbiota dysbiosis through pre-/probiotics may be a novel therapeutic target for this untreated disease. However, several limitations need to be taken into account in our study. Firstly, the sample size in our study was small due to the low morbidity and rapidly progressive process of MSA (Geser et al., 2006; Fanciulli and Wenning, 2015). Thus, the larger-scale researches including different population were required. Then, our study was a cross-sectional study and whether the change of gut microbiota was the initiating stimulator or the result of MSA pathogenesis remained unclear. Therefore, investigation on the gut microbiota in longitudinal studies was indispensable across different periods of MSA process.

## Conclusion

In conclusion, we found altered gut microbiota composition and functional pathways in MSA patients. Our study provided microbial basis for the further multi-omics research and functional experiments to clarify roles of gut microbiota in MSA, which may improve our understanding of MSA pathogenesis and facilitate the development of accurate diagnosis methods, as well as novel therapeutic strategies targeting modifying gut microbiota in MSA patients.

## Data Availability Statement

The raw data supporting the conclusions of this manuscript are available in the SRA database under the accession number PRJNA532538.

## Ethics Statement

This study was approved by the Ethics Committee of Xiangya Hospital of Central South University in China. All subjects gave written informed consent in accordance with the Declaration of Helsinki.

## Author Contributions

LW, XZ, and HJ designed the project. LW, XZ, CW, ZC, HP, XuH, YP, PW, and TL organized and executed the experiment. LW, XZ, HY, YS, XiH, KXu, YX, and LH analyzed and interpreted the data. LW wrote the manuscript. KXi, BT, and HJ revised the manuscript. HJ agreed to be accountable for the content of the work. All authors approved the final version of the manuscript.

## Conflict of Interest

The authors declare that the research was conducted in the absence of any commercial or financial relationships that could be construed as a potential conflict of interest.
