# Corrigendum: Alterations of the Gut Microbiota in Multiple System Atrophy Patients

**DOI:** 10.3389/fnins.2020.00019

**Published:** 2020-01-31

**Authors:** Linlin Wan, Xin Zhou, Chunrong Wang, Zhao Chen, Huirong Peng, Xuan Hou, Yun Peng, Puzhi Wang, Tianjiao Li, Hongyu Yuan, Yuting Shi, Xiaocan Hou, Keqin Xu, Yue Xie, Lang He, Kun Xia, Beisha Tang, Hong Jiang

**Affiliations:** ^1^Department of Neurology, Xiangya Hospital, Central South University, Changsha, China; ^2^National Clinical Research Center for Geriatric Disorders, Central South University, Changsha, China; ^3^Key Laboratory of Hunan Province in Neurodegenerative Disorders, Central South University, Changsha, China; ^4^Laboratory of Medical Genetics, Central South University, Changsha, China; ^5^Department of Neurology, Xinjiang Medical University, Urumchi, China

**Keywords:** multiple system atrophy, microbiota, metagenomics, functional pathways, inflammation

There is an error in the Funding statement. The correct number for the National Natural Science Foundation of China is No. 81771231, 81471156 to HJ and No. 81600995 to YS.

We also neglected to include the funder **the National Natural Science Foundation of China**, **No. 81974176 to HJ, 81901169 to ZC, No. 81901305 to CW**.

The final funding part is as follows:

This study was supported by the National Key Research and Development Program of China (Nos. 2016YFC0901504 and 2016YFC0905100 to HJ and No. 2016YFC1306000 to BT), the National Natural Science Foundation of China (Nos. 81771231, 81471156, and 81974176 to HJ, No. 81600995 to YS, No. 81901169 to ZC, and No.81901305 to CW), the Key Research and Development Program of Hunan Province (No. 2018SK2092 to HJ), the Scientific Research Foundation of Health Commission of Hunan Province (No. B2019183 to HJ), and the Clinical and Rehabilitation Fund of Peking University Weiming Biotech Group (No. xywm2015I10 to HJ).

The authors apologize for this error and state that this does not change the scientific conclusions of the article in any way. The original article has been updated.

