# Supplementary material for: Alterations of the Gut Microbiota in Multiple System Atrophy Patients
Source: Front Neurosci. 2019 Oct 18;13:1102. doi: 10.3389/fnins.2019.01102 (PMC6813281; doi:10.3389/fnins.2019.01102)
Supplement: Supplementary file 1 [file Data_Sheet_1.PDF]

Supplementary Material

Supplementary Figures

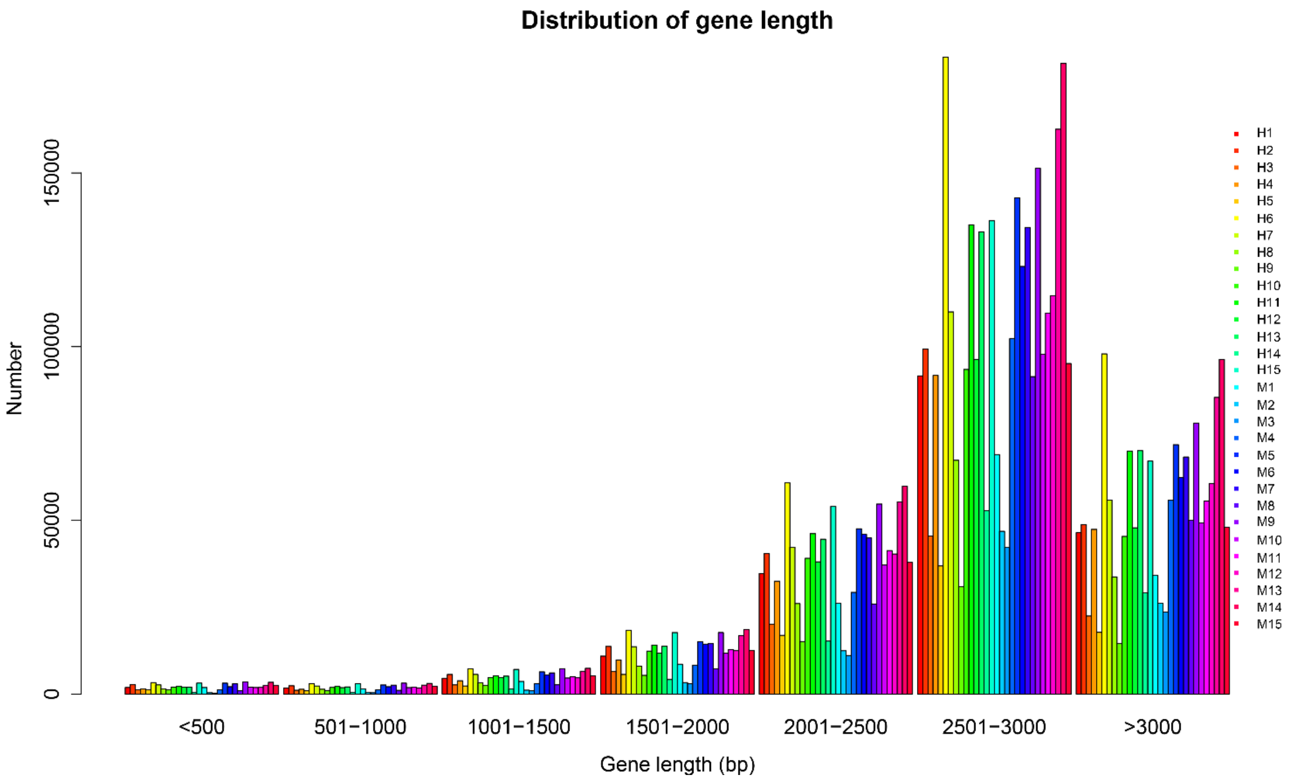

Supplementary Figure 1. The distribution of gene length in each sample generated through gene prediction. Different color represented different sample. H, healthy group; M, MSA group.

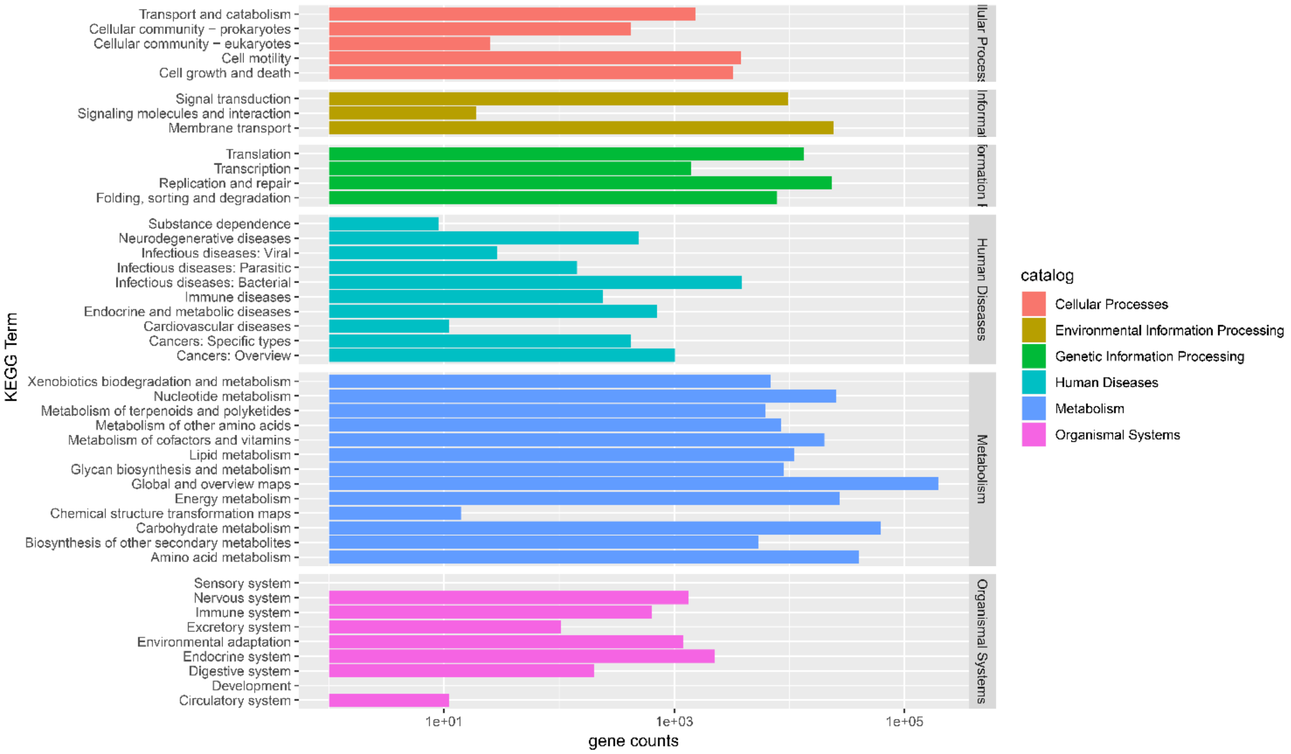

Supplementary Figure 2. The distribution of gene counts under the different functional pathways of KEGG database. The functional catalogs were Cellular Process, Environmental Information Processing, Genetic Information Processing, Human Disease, Metabolism and Organism Systems from the top down.

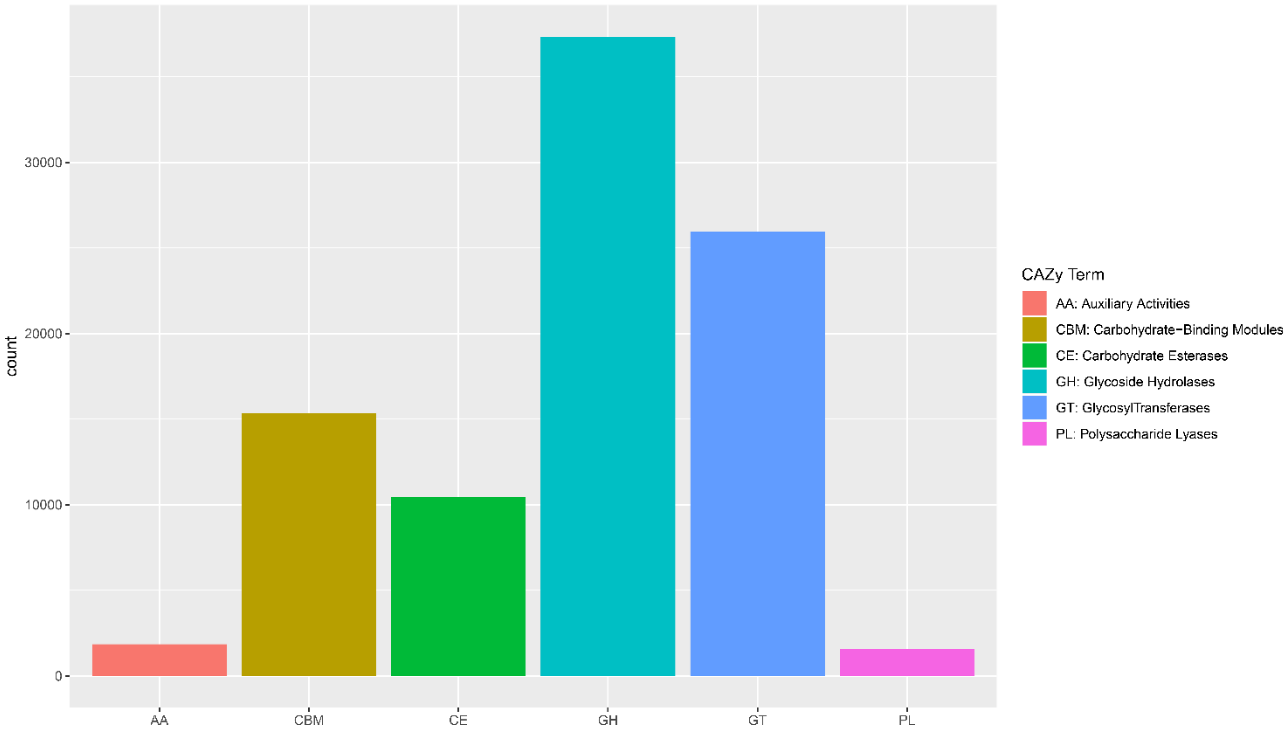

Supplementary Figure 3. The distribution of gene counts under the different Cazymes terms. The terms were Auxiliary Activities (AA), Carbohydrate-Binding Modules (CBM), Carbohydrate Esterases (CE), Glycoside Hydrolases (GH), Glycosyl Transferases (GT) and Polysaccharide Lyases (PL) from left to right.
